# Improving the Care of Severe, Open Fractures and Postoperative Infections of the Lower Extremities: Protocol for an Interdisciplinary Treatment Approach

**DOI:** 10.2196/57820

**Published:** 2024-09-16

**Authors:** Steffen Rosslenbroich, Marion Laumann, Joachim Hasebrook, Sibyll Rodde, John Grosser, Wolfgang Greiner, Tobias Hirsch, Stefan Windrich, Michael J Raschke

**Affiliations:** 1 Klinik und Poliklinik für Unfall-, Hand- und Wiederherstellungschirurgie University Hospital Muenster Muenster Germany; 2 ZEB Business School Steinbeis University Madgeburg Germany; 3 Fakultät für Gesundheitswissenschaften University Bielefeld Bielefeld Germany; 4 Fachklinik Hornheide Muenster Germany; 5 FuE Bereich Gesundheit | R&D Division Health OFFIS e.V. - Institut für Informatik Oldenburg Germany

**Keywords:** open fracture, open soft tissue damage, telemedicine, plastic surgery, infectiology, limb function, health-related quality of life, workload, work engagement, health economic evaluation

## Abstract

**Background:**

Patients with open fractures often experience complications during their injury. The treatments incur high costs. Interdisciplinary cooperation between different medical disciplines may improve treatment outcomes. Such cooperation has not yet been envisaged in the German health care system.

**Objective:**

The aim of the study is to improve the treatment of fractures with open soft tissue damage or postoperative complications in terms of duration and sustainability in a region in northwest Germany. Largely standardized diagnostics and therapy are intended to optimize processes in hospitals. In addition, a reduction in the duration of treatment and treatment costs is to be achieved.

**Methods:**

Using a digital platform, physicians from 31 hospitals present patient cases to an interdisciplinary group of experts from the fields of plastic surgery, infectiology, hygiene, and others. The group of experts from the environment of the University Hospital Münster promptly makes a joint treatment recommendation for the individual case. The plan is to examine 3300 patients with open fractures or surgical complications. As consortium partners, there are also 3 statutory health insurance companies. The extent to which the therapy recommendations are effective and contribute to cost reduction in the health care system will be empirically investigated in a stepped-wedge cluster-randomized design. In addition, medical and nonmedical professional groups involved in the project will be asked about their work in the project (in total, 248 clinic employees). The primary outcome is the complication rate of open fractures or the occurrence of postoperative complications. As secondary outcomes, the number of antibiotics administered, limb function, and quality of life will be assessed. The health economic evaluation refers to the costs of health services and absenteeism. For the work-related evaluation, workload, work engagement, work-related resources, readiness for technology, and ergonomic aspects of the new telemedical technology will be collected. In addition, clinic employees will give their assessments of the success of the project in a structured telephone interview based on scaled and open-ended questions.

**Results:**

The project started in June 2022; data collection started in April 2023. As of mid-June 2024, data from 425 patients had been included. In total, 146 members of staff had taken part in the questionnaire survey and 15 had taken part in the interviews.

**Conclusions:**

Standardized treatment pathways in the standard care of patients with open fractures and postoperative infections will be established to reduce complications, improve chances of recovery, and reduce costs. Unnecessary and redundant treatment steps will be avoided through standardized diagnostics and therapy. The interdisciplinary treatment perspective allows for a more individualized therapy. In the medium term, outpatient or inpatient treatment centers specialized in the patient group could be set up where the new diagnostic and therapeutic pathways could be competently applied.

**Trial Registration:**

German Clinical Trials Register DRKS00031308; https://drks.de/search/de/trial/DRKS00031308

**International Registered Report Identifier (IRRID):**

DERR1-10.2196/57820

## Introduction

### Background

Open fractures of the lower extremities are common, occurring with an incidence of approximately 11.5 per 100,000 [1]. Patients with open fractures often experience complications in the course of the disease, such as soft tissue infections, bone inflammation, defective bone fuse, and increased tissue pressure around the injury, with negative effects on blood supply and the nervous system [2,3]. Even for highly experienced service providers, it is often not possible to provide a sufficient quality of care for disciplines such as trauma, vascular and plastic surgery, infectiology, microbiology, and hygiene [4]. However, such interdisciplinary treatment has not yet been established in German primary and standard care. Currently, many patients with complicated or incorrectly healed fractures, infections, or chronically open wounds see several physicians in different hospitals, combined with inpatient transfers, redundant diagnostics, and long sick leave [5-7]. In many cases, the healing process is significantly impaired, and it is not uncommon for serious restrictions to remain.

In addition to the physical and psychological consequences for those affected, there are also the considerable costs of the health care system [1,8,9]. Patients with open lower-leg fractures incur almost twice as much cost (€11,000 [US $12,000]) as patients with closed fractures (€6600 [US $7100]) [5]. Open fractures with soft tissue infections lead to increased antibiotic use, additional surgeries, and longer hospital stays, which can increase costs 6-fold [5,10,11]. Against the backdrop of demographic change [12,13] as well as the increasing incidence of fractures due to comorbidities (eg, osteoporosis, gait uncertainty, and neurological diseases) and complications in the healing process from the age of 65 years onward, improving treatment options for complicated fractures and reducing the complication rate are of extreme importance [14]. Main complications that are encountered in this context are pseudarthrosis and chronic osteomyelitis. Pseudarthrosis is defined as a problem in fracture healing with missing consolidation of >6 months after trauma. Chronic osteomyelitis is a state in which destructive changes within the bone occur due to an infectious process. Both conditions prolong the healing process and usually make several surgical procedures necessary. In some cases, even an amputation must be considered.

In the United Kingdom, treatment structures have already been put in place to provide adequate and timely care to patients with these kinds of injuries. The British Orthopaedic Association and the British Association of Plastic, Reconstructive, and Aesthetic Surgeons introduced evidence-based standards of care for the treatment of severe, open lower-leg fractures (British Orthopaedic Association Standards for Trauma and Orthopaedics) [15]. It has been demonstrated that an early transfer of patients to specialized trauma centers and cooperation between surgical and nonsurgical disciplines significantly improve the course of treatment. Studies show a significant reduction in the infection rate, fewer additional surgeries, more successful limb reconstructions, and a reduction in treatment time [16-20].

Thus, concrete guidelines for the treatment of complicated fractures with soft tissue damage help not only improve the chances of treatment and healing but also reduce the costs of medical care. In particular, the involvement of an interdisciplinary team is essential for high-quality and cost-effective care [21]. In a joint interdisciplinary case conference, information and expertise can be exchanged, and treatment recommendations can be made based on broad expertise. This saves time and reduces redundant examinations and collisions of different treatment recommendations.

To consolidate treatment competence and ensure treatment quality, the standardization of specifications as well as diagnostic and treatment-related processes is essential. In the field of cancer therapy, interdisciplinary therapy recommendations have been established for decades within the framework of tumor boards. After intensive, interdisciplinary expert discussions, such a board makes a binding, patient-specific treatment recommendation considering all disease-relevant aspects [22].

So far, there is nothing comparable in Germany for the treatment of trauma to the extremities. In some large centers, there are interdisciplinary structures that deal with complex treatment situations. However, there is still no established process for the regular implementation of therapy steps. To date, there is also no way to standardize postoperative complications and treat them using stringent treatment pathways. Currently, people who have an open fracture or, in this context, a postoperative complication are admitted to the primary care clinic and treated on a monodisciplinary basis. The healing process of complicated fractures is further complicated by the fact that there are no contact points that take over the management of further treatment when the treatment options of a clinic for primary and standard care have been exhausted.

### Objectives

The aim of the EXPERT (Extremity Boards for process optimization, evaluation, risk minimization, and therapy optimization for fractures with soft tissue damage or postoperative infection of the lower extremities in the trauma network) project is to improve the treatment of fractures with open soft tissue damage or postoperative complications in northwest Germany rapidly and sustainably. Reductions in the duration and costs of treatment are further aims.

To achieve this, an interdisciplinary group of medical experts with a wide range of university expertise (eg, plastic surgery, infectiology, and hygiene) will make joint treatment recommendations within the framework of video case presentations. Consortium partners are 3 German statutory health insurance companies (BARMER, Techniker Krankenkasse, and AOK NordWest; see also Multimedia Appendix 1).

Physicians in standard care hospitals present their cases to the expert group (the Extremity Board) via telemedicine software and receive treatment recommendations tailored to the respective patients in a timely manner. In this way, the treatment result can be improved by adapting the individual therapy at an early stage.

On the basis of the knowledge gained in the Extremity Board, the interdisciplinary specialist group will define guidelines during the project to objectify decision-making paths, streamline processes, and define therapy algorithms. Through the close cooperation of the expert group with hospitals of primary and standard care, a joint agreement can be reached on treatment and documentation standards that relate to both the care of acute symptoms and postoperative complication management. The introduction of a web-based software platform enables both a secure exchange of medical information across the boundaries of different professional groups and the provision of interdisciplinary expertise in rural, structurally weak regions and regions affected by a shortage of specialists.

The effects of the new form of care based on the interdisciplinary approach and the associated standardization of diagnostics and therapy will be scientifically evaluated. Patient-related clinical success criteria as well as economic outcomes will be collected. As the new form of care has both content-related and organizational effects on the work in the clinics, work-related aspects will also be examined.

### Research Questions and Hypotheses

#### Clinical and Health Economic Hypotheses

It is assumed that efficient telemedical access to an interdisciplinary expert forum (the Extremity Board) with simultaneous therapy decisions in the treatment of fractures with open soft tissue damage and postoperative complications will achieve significant reductions in the length of inpatient stay, the total duration of treatment, and the complication and reoperation rate [23]. The measures optimize therapy pathways, standardize them, and make them more cost-effective. The establishment of the Extremity Board leads to higher guideline adherence, standardization, and documentation quality and, consequently, to reduced antibiotic consumption and a reduction in unnecessary diagnostics.

#### Work-Related Hypotheses

For the ergonomic evaluation of the EXPERT project, the job demands–resources (JD-R) model [24-26] on the influence of work resources and work requirements on work behavior will be used. In this theoretical model, which is backed up by a wide range of findings [27-29], work resources and work requirements are considered to be independent factors influencing work engagement and perceived stress from work. High cognitive, emotional, and physical demands such as time pressure, workload, complexity of tasks, and emotional stress lead to high demands, which has long-term negative consequences for employees and the company (eg, employee burnout, low job satisfaction, and low productivity [30]).

On the other hand, work resources such as social support, feedback, autonomy, and meaningfulness have a motivating effect because they satisfy basic needs for self-efficacy, attachment, security, and control [31,32]. Therefore, diverse work resources lead to more work engagement, which has positive consequences for employees and the company (eg, high job satisfaction and high productivity [30]).

The JD-R model also postulates that work requirements and work resources interact with each other, which affects the perception of stress and motivation. Thus, the availability of resources can mitigate the negative impact of the requirements. Resources seem to have a positive effect on motivation and work engagement, especially when work demands are high [33]. When work demands are high, employees increasingly perceive existing resources as helpful and useful, which helps them cope with the demands. This results in a subjectively lower burden (coping hypothesis [34,35]).

**Figure 1:**
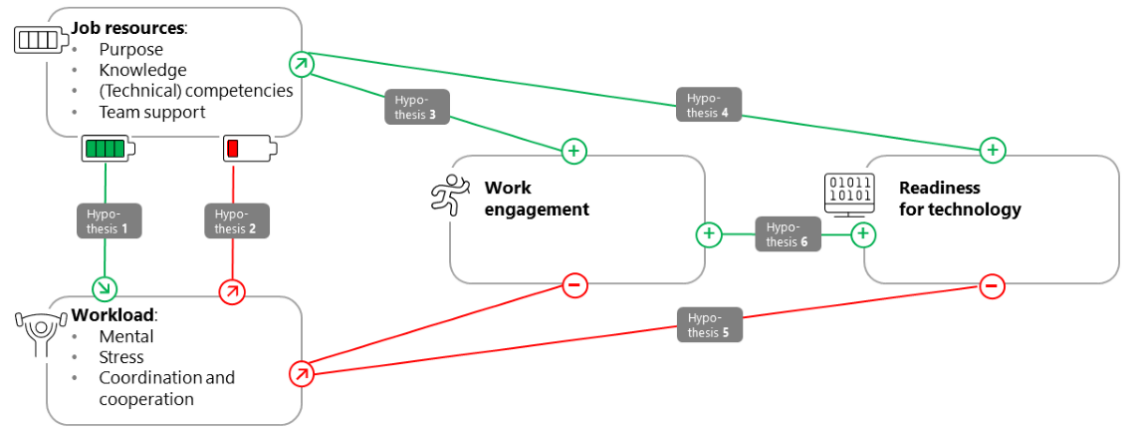
Overview of work-related hypotheses concerning the relationship among work resources, workload, work engagement, and readiness for technology.

On the basis of the theoretical assumptions and empirical findings, the following hypotheses can be derived for the EXPERT project (Figure 1): (1) work requirements and workload increase in the intervention phase of the EXPERT project (hypothesis 1a); (2) work-related resources such as knowledge, competencies, and team support increase during the intervention phase (hypothesis 1b); and (3) the technical competence of employees increases during the intervention phase (hypothesis 1c).

Above- or below-average resources are recorded with the median of differences (pretest-posttest estimates of resources), leading to other hypotheses: (1) above-average resources are associated with lower workload, and below-average resources are associated with higher workload (hypothesis 2); (2) above-average resources are associated with higher work engagement (hypothesis 3a); (3) when resources increase, work engagement increases (hypothesis 3b); (4) if resources increase (exclusion of technical competence as a resource), then readiness for technology increases while the burden decreases (hypothesis 4a); (5) as technical competence (resource) increases, the burden decreases (hypothesis 5a); (6) when technical competence (resource) decreases, the burden increases (hypothesis 5b); and (7) above-average work engagement leads to higher readiness for technology, and below-average work engagement leads to lower readiness.

## Methods

### Study Design

#### Clinical and Health Economic Evaluation

For the organization and evaluation of the new form of care, a stepped-wedge design was chosen [36,37]. In a stepped-wedge design, there are different clusters that start recruitment at the same time in the control phase. The individual clusters then move gradually into the intervention phase in randomized cohorts. In the EXPERT project, there is a transition phase between the control and intervention periods, which serves to implement and test the new form of care in the individual clinics as well as train the staff.

**Figure 2:**
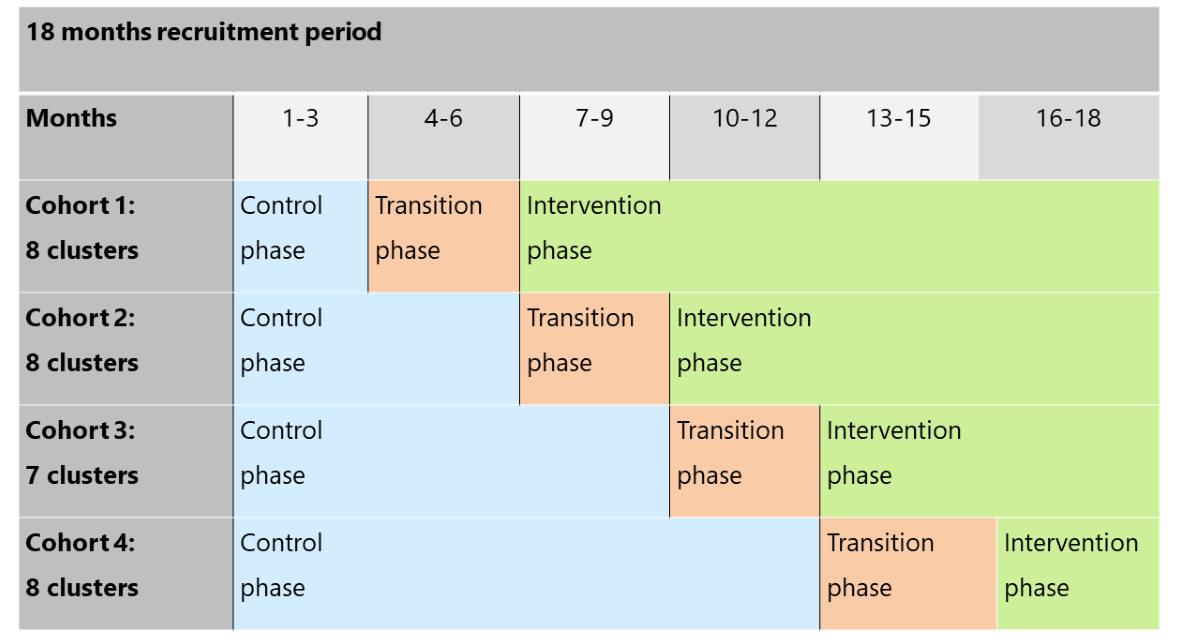
Representation of the stepped-wedge design in the EXPERT (Extremity Boards for process optimization, evaluation, risk minimization, and therapy optimization for fractures with soft tissue damage or postoperative infection of the lower extremities in the trauma network) project.

Randomization in the EXPERT project will take place at the hospital level. There are 31 hospitals participating in the study. Each clinic forms a cluster that belongs to 1 of 4 cohorts. The clusters within a cohort always enter the next study phase (transition or intervention phase) at the same time. The study will be conducted over a period of 24 months (18-month recruitment period+6-month follow-up of the last enrolled person). Data collection regarding the individual course of treatment takes place from the time of enrollment in the patient sample until 6 months later. Figure 2 shows the planned course of the study in the stepped-wedge design.

#### Work-Related Evaluation

For the ergonomic evaluation, the “difference-in-difference” method will be used within the stepped-wedge design [38]. This methodological approach includes a treatment group and a control group, each of which is examined at 2 measurement points (t0 and t1). In the treatment group, but not in the control group, an intervention takes place between the measurement points. The first difference that is measured refers to a possible change from measurement time t0 to measurement time t1. This is calculated for both the treatment and control groups. The second difference is the difference between these 2 differences (ie, the difference within the control group is subtracted from the difference within the treatment group). The result can be interpreted as a causal effect of an intervention [38,39].

In the EXPERT project, the clinics that have not yet implemented the project measures serve as a control group, which will be evaluated at the same time as the clinics that are already implementing the new measures. Cohorts 1 and 2 are the treatment group, whereas cohorts 3 and 4 act as the control group.

### Data Sampling

#### Clinical and Health Economic Evaluation

Studies show that, in approximately 40% of cases, complications occur during the treatment in open fractures [40]. The new form of care is realistically intended to reduce the complication rate to 30%. A type-1 error (α) of 5%, a type-2 error (β) of 20% (80% power), and an intraclass correlation coefficient of ρ=0.05 are assumed [36]. Considering the transition phase and a dropout rate of 20%, the total number of cases required to demonstrate a treatment effect is 3366 patients (of whom n=1377, 40.91% would be in the control phase; n=1428, 42.42% would be in the intervention phase; and n=561, 16.67% would be in the transition phase). This equates to 17 patients per hospital per 3-month interval.

Only patients who can give consent and who have statutory health insurance will be included in the project and will give their written consent to participate and share their data in the use of telemedicine. The study will include patients with fractures of the extremities with open soft tissue damage (according to Gustilo et al [41]) or with postoperative complications (according to the criteria of the Association of the Study of Internal Fixation Anti-Infection Task Force) or people with unplanned resurgery within 6 months of initial surgery. Specifically, these are *International Statistical Classification of Diseases and Related Health Problems, 10th Revision* (*ICD-10*), diagnoses [42] related to open soft tissue damage in fractures or dislocations, amputations of the lower extremity, infections of joints or joint endoprostheses of the lower extremities [43], and pseudarthrosis and delayed fracture healing of the lower extremity (see the list in Textbox 1).

Patients who are not capable of giving consent, who do not give their consent to participate or to data transfer, or who do not have statutory health insurance will be excluded from the study. Also excluded will be persons to whom the aforementioned *ICD-10* diagnoses do not apply (ie, who do not have a fracture of the extremities or extremities or have a closed fracture of the extremities without open soft tissue damage and who do not experience a postoperative complication within 6 months of initial surgery). The inclusion and exclusion criteria are summarized in Textbox 2.

***International Statistical Classification of Diseases and Related Health Problems, 10th Revision* (*ICD-10*), diagnoses to be included in the EXPERT (Extremity boards for process optimization, evaluation, risk minimization, and therapy optimization for fractures with soft tissue damage or postoperative infection of the lower extremities in the trauma network) study.**

***ICD-10* code and description of diagnosis**

- S71.87: grade-I soft tissue damage due to open fracture or dislocation of hip and thigh
- S71.88: grade-II soft tissue damage due to open fracture or dislocation of hip and thigh
- S71.89: grade-III soft tissue damage due to open fracture or dislocation of hip and thigh
- S81.87: grade-I soft tissue damage due to open fracture or dislocation of lower leg
- S81.88: grade-II soft tissue damage due to open fracture or dislocation of lower leg
- S91.88: grade-II soft tissue injury due to open fracture or dislocation of foot
- S78: traumatic amputation of hip and thigh
- S88: traumatic amputation of lower leg
- S98: traumatic amputation of upper ankle and foot
- T84.6: infection and inflammatory reaction due to internal osteosynthesis device (any location)
- T84.7: infection and inflammatory reaction from other orthopedic endoprostheses, implants, or grafts

**Inclusion and exclusion criteria.**

**Formal criteria**

- Inclusion criteria
  Consent to study participation and data transfer
  Legal capacity to consent to study participation
  Statutory health insurance
- Exclusion criteria
  Lack of consent to study participation and data transfer
  Unclear legal capacity or capacity to consent
  Not covered by statutory health insurance

**Clinical criteria**

- Inclusion criteria
  Patients who meet at least one of the following 2 criteria: fractures of the extremities with open soft tissue damage (according to Gustilo et al [41]) OR postoperative complications (according to the criteria of the Association of the Study of Internal Fixation Anti-Infection Task Force) or unplanned reoperation within 6 months of initial surgery; these criteria are operationalized via the *International Statistical Classification of Diseases and Related Health Problems, 10th Revision*, codes for the following: open soft tissue damage in fractures or dislocations as well as amputations of the lower extremities; infections of the lower extremities (excluding joints and joint arthroplasty); infections of joints or joint endoprostheses of the lower extremities; pseudarthrosis and delayed fracture healing of the lower extremities
- Exclusion criteria
  Patients who meet both of the following criteria: no fracture of the extremities or closed fracture of the extremities without open soft tissue damage AND no postoperative complication or unplanned reoperation within 6 months of initial surgery

#### Work-Related Evaluation

Physicians and nurses who are or will be involved with the project take part in the work-related evaluation. With medium to small effect sizes (η^2^>0.05 and <0.15), a type-1 error (α) of 5%, and a type-2 error (β) of 20% (80% power), a minimum number of 120 people per measurement point is required for the work- and project-related evaluation (60 people each in the control and treatment groups). A total of 4 employees per clinic at measurement time t0 and 4 employees at measurement time t1 are included in the sample. This results in 31 × 8 = 248 elevations. Care is taken to interview physicians and nurses in equal parts, all of whom are or will be involved in the project measures.

Regarding the interviews for the assessment of the objectives and project implementation, 2 persons from the nursing service and 2 persons from the medical profession are to be interviewed at time t1 in the clinics that are already implementing the new form of care. Approximately 72 people from at least 18 clinics will take part in the interviews.

### Ethical Considerations

Ethics approval has been granted by the ethics committee of the Medical Association of Westphalia-Lippe under the number 2023-067-f-S. This study is conducted per the tenets of the Declaration of Helsinki. All data have been collected, anonymized, analyzed, and stored in accordance with German General Data Protection Regulation legislation based on the European General Data Protection Regulation. The accordance to these data protection standards is approved and enforced by OFFIS-Institute for Information Technology, an institute related to the University of Oldenburg specializing on research services and data protection.

Participation is voluntary, and participants are required to provide oral and written consent to take part before entering the study. All participants received oral and written information before signing a consent form upon enrollment in the trial, and no financial compensation was made to participants in the study. The protocol was registered with the German Clinical Trials Register database (DRKS00031308).

### Outcomes and Measures

#### Clinical and Health Economic Evaluation

The primary outcome and various secondary clinical outcomes are recorded based on data from the clinical case file as well as routine data from the health insurance companies.

The primary clinical outcome is the complication rate of open fractures or the occurrence of postoperative complications, which is collectively defined as a dichotomous outcome. Either the occurrence or nonoccurrence of complications is observed. The following events are complications if they occur within 6 months of enrollment in the sample: (1) occurrence of pseudarthrosis (incomplete fracture healing; *ICD-10* code M96.0); (2) occurrence of chronic osteomyelitis (*ICD-10* code M86.69); (3) unplanned follow-up procedures, such as wound revisions and Negative Pressure Wound Therapy (*ICD-10* code S81.801); (4) deviation from standardized antibiotic therapy (extension of the duration of therapy or change or expansion of the active substance; *ICD-10* code T36.95); (5) occurrence of antibiotic-associated complications (eg, clostridia infection or allergy; *ICD-10* code B96.7); (6) infection with resistant germs (eg, methicillin-resistant *Staphylococcus aureus*) that occurred during inpatient treatment and did not exist at the time of trauma (*ICD-10* code A49.02); (7) need for unplanned inpatient treatment; and (8) death of the patient related to the trauma (not due to other causes).

Secondary clinical outcomes include the number of antibiotics administered, limb function, and health-related quality of life (HRQoL) [44,45].

Health economic outcomes are mapped through claims data from health insurance companies. These will be recorded for each patient included in the project up to 6 months after enrollment in the sample. Specifically, the following features will be recorded: (1) use of health services (eg, contact with physicians and therapists, operations, and inpatient stays), (2) cost of health care services (inpatient hospital costs, outpatient costs, rehabilitation costs, medical and medical aid costs, drug costs, and total costs), (3) number of days on which there is incapacity for work, and (4) number of days on which sickness benefits are received.

Table 1 provides an overview of the clinical and health economic outcomes used in this study.

Patients’ HRQoL is assessed at the time of enrollment and 6 months later using two internationally used measuring instruments: (1) the German-language version of the royalty-free Veterans RAND 36-item Health Survey [44] and (2) the German-language version of the EQ-5D of the EuroQol Group [45,46].

The Veterans RAND 36-item Health Survey is a generic profile questionnaire and measures HRQoL regarding the dimensions “Physical Functioning,” “Role-Physical,” “Role-Emotional,” “General Health,” “Social Functioning,” “Vitality,” “Bodily Pain,” and “Mental Health.”

The EQ-5D as a generic index instrument contains the 5 dimensions “Mobility,” “Self-care,” “Usual activities,” “Pain/Discomfort,” and “Anxiety/depression” as well as a visual analog scale. The results of the EQ-5D can be used to create a health profile as well as calculate an index value [46]. As an instrument validated in Germany, the index for HRQoL recorded using the EQ-5D is particularly well suited as a health economic outcome measure [46].

**Table 1:** Clinical and health economic outcomes.

Outcome					Data source			Measurement time
**Primary outcome**								
	**Complication rate, defined as the occurrence of at least 1 of the following events within 6 months of trauma [42]:**			Clinical and claims data			6 months	
		Occurrence of pseudarthrosis (incomplete fracture healing)	Clinical data			6 months		
		Occurrence of chronic osteomyelitis	Clinical data			6 months		
		Unplanned follow-up procedures, such as wound revisions and NPWT^a^	Clinical and claims data			6 months		
		Deviation from standardized antibiotic therapy (extension of the duration of therapy or change or expansion of the active substance)	Clinical and claims data			6 months		
		Occurrence of antibiotic-associated complications (eg, clostridia infection or allergy)	Clinical and claims data			6 months		
		Infection with resistant germs (eg, MRSA^b^) that occurred during inpatient treatment and did not exist at the time of trauma [43]	Clinical data			6 months		
		Need for unplanned inpatient treatment	Clinical and claims data			6 months		
		Death of the patient related to the trauma (not due to other causes)	Clinical and claims data			6 months		
**Secondary clinical outcomes**								
	Number of antibiotics administered			Clinical and claims data			6 months	
	Limb function			Clinical data			6 months	
	HRQoL^c^, measured using the VR-36^d^ [44] and EQ-5D [45,46]			Clinical data			0 and 6 months	
**Health economic outcomes**								
	Use of health care services			Claims data			6 months	
	Cost of health care services			Claims data			6 months	
	Days of incapacity for work			Claims data			6 months	
	Sickness benefit days			Claims data			6 months	

^a^NPWT: negative pressure wound therapy.

^b^MRSA: methicillin-resistant *Staphylococcus aureus*.

^c^HRQoL: health-related quality of life.

^d^VR-36: Veterans RAND 36-item Health Survey.

#### Work-Related Evaluation

##### Questionnaires

Building on experiences and results from previous ergonomics studies in the fields of medical care and outpatient and inpatient care [47-49] and using the JD-R model as a theoretical frame of reference, the work-related evaluation uses validated measurement tools that measure workload, work engagement, work-related resources, and technological readiness. As the new form of care in the EXPERT project requires a competent application of telemedical technology, ergonomic aspects of the new telemedicine application are also recorded. The 7 items for the usability of the telemedicine application are only administered to the treatment group at the second measurement time. Therefore, the EXPERT questionnaire comprises 4 instruments with 31 items without ergonomic aspects and 5 instruments with 38 items with ergonomic aspects. Empirical comparative values are available for all measuring instruments used.

Workload is measured using the National Aeronautics and Space Administration Task Load Index [50] in a version for teamwork [51,52]. For this study, an abridged version of this questionnaire with 8 items in the German translation is used. On a scale of 1 to 10 (1=*very low*; 10=*very high*), employees estimate the mental, time, and performance-related work requirements as well as the effort regarding exchange, organization of work, team effectiveness, and mutual support for the past 3 months and give an assessment of their satisfaction with the cooperation. For example, a formulation is “How much effort was required to organize the work (e.g., coordination of who takes on which task, when which task is completed)?”

Work engagement is measured using the ultra–short form of the Utrecht Work Engagement Scale, which contains 3 items [53]. The German translations of “At work I am full of energy,” “I am enthusiastic about my work,” and “I am completely absorbed in my work” are used, which are rated on a scale from 0 to 6 (0=*never*; 6=*always*).

Work resources are measured using the German-language questionnaire on resources and requirements in the world of work [54]. For the EXPERT project, 11 items are used, which relate to the resources “Leadership Support,” “Feedback,” “Peer-Support,” “Transparency,” “Diversity,” “Meaningfulness,” “Role Clarity,” “Learning Opportunity,” “Knowledge Management,” “Team Atmosphere,” and “Qualification.” All items are formulated as statements (eg, “I will be informed about all important content when implementing the new measures” [“transparency” resource]), which are rated on a scale from 1 to 6 (1=*not at all true*; 6=*completely true*).

Affinity for technology is measured using the German-language short scale for measuring readiness for technology [55]. It is a questionnaire with 12 items in the 3 scales “TB acceptance,” “TB control belief,” and “TB competence,” of which 9 items are used in this study. The item formulations have been adapted to the EXPERT project (eg, “I am very interested in the new telemedicine application” [acceptance scale], “It essentially depends on me whether I am successful in using the new telemedicine application” [control conviction scale], and “For me, dealing with technical innovations is usually too much to handle” [competence scale]).

The software ergonomics of the new telemedicine application are assessed using the German-language questionnaire Norm of the International Standard Organization 9241/10 [56,57]. On the basis of this instrument, software is evaluated regarding “adaptivity to requirements,” “comprehensibility,” “task appropriateness,” “ability to describe itself,” “fault tolerance,” “individualizable,” and “learning conduciveness.” The assessment of the 7 items is made on a bipolar scale based on symbols (from “– – –” to “+++”).

Finally, employees provide information about their profession (physician or nurse), whether they hold a management position (yes or no), and whether they already had experience in the use of telemedicine before the EXPERT project (yes or no).

##### Interviews

The structured telephone interview on the implementation of the project comprises both predetermined and open-ended questions, the content of which is based on the planned course of the EXPERT project. For each of the 4 project phases—“Preparation,” “Development of new standards,” “Cooperation with the Extremity Board,” and “Implementation of therapy recommendations”—3 to 5 items were designed in the form of statements, which are assessed by the interviewees based on a 10-point scale. For example, one item regarding the project phase “Preparation” is as follows: “All employees involved in the project were able to obtain sufficient information about the new work steps and tasks, e.g., through training. (1=not true at all, 10=completely true).” An example for the project phase “Collaboration with the Extremity Board” is as follows: “The case presentations in the Extremity Board are helpful for the planning of treatments.”

The open questions of the interview relate to aspects that the interviewees consider to be particularly important for the transfer of the project measures into standard care. The questioning technique used in this study is a simplified modification of the repertory grid technique [58,59]. In total, 2 open-ended questions refer to sequencing of steps within the project: (1) preparation phase, (2) development of new standards, (3) cooperation with the Extremity Board, and (4) implementation of the therapy recommendations. The participants are asked to describe what they like or dislike within the project steps. Regarding the most likeable and the most undesirable aspects, the participants are asked how important these aspects are for the future implementation of an Extremity Board (1=*not important at all*; 10=*very important*). This results in 8 aspects per interview that are relevant for the long-term implementation of the Extremity Board from the point of view of the hospital staff.

### Data Analysis

#### Clinical and Health Economic Evaluation

The eligible patients are enrolled in the project by trained staff. Patients are informed about the objectives and course of the project and give their written consent to participate. Each case is summarized with all relevant information (eg, anamnesis, findings, and imaging) in a clinical case file using data-secure web-based software. The case files are checked for completeness and, after approval, transmitted digitally to the interdisciplinary expert group, the Extremity Board. This group of experts is recruited from the following disciplines: trauma surgery, plastic surgery, vascular surgery, radiology, angiology, and antibiotic stewardship (microbiology, hygiene, infectiology, and pharmacy) [43]. The experts meet at least weekly via web conference and, depending on the urgency, discuss the cases together. Agreed interdisciplinary therapy recommendations are written and transmitted back to the presenting clinic via the web-based software platform. In addition, guidelines will be defined in the course of the project on the basis of the findings gained by the Board to objectify decision-making paths, streamline processes, define therapy algorithms, and enable the supraregional applicability of these standards.

The clinical case files of all patients included in the EXPERT project will be merged with the respective patient-related health insurance data in an anonymized data file for each case while maintaining data protection. Claims data from health insurance companies provide information about the use of health services and the costs that arise in doing so. To obtain information about the individual course of treatment after the introduction of the new form of care, claims data for the following 6 months are included in the evaluation for each patient from the time of enrollment in the sample. Thus, the clinical case file and the associated health insurance data depict the course of treatment of a patient, which is included in the evaluation.

To be able to evaluate the extent to which the new care measures improve the chances of recovery and reduce costs, various clinical and economic outcomes are collected.

First, the primary outcome of the complication rate as well as the secondary and health economic outcomes are evaluated descriptively using absolute and relative frequency distributions as well as position and scattering measures (eg, median, variances, SD, and representation by means of box plots or histograms). The differences between patients in the control and intervention phases are checked using appropriate statistical methods (eg, Mann-Whitney *U* test [60,61], chi-square test, or odds ratio [62]). In addition, individual subgroups (eg, by sex and age) are analyzed to check the data for possible biases.

This descriptive analysis is followed by interference statistical analyses. By means of suitable regression analyses (eg, using generalized linear regression models), it can be examined which regressors have an influence on the primary and secondary outcomes. In addition to the consideration of temporal and intervention-related effects, the influence of potential confounders is also controlled for. This is because various patient- and trauma-associated factors are relevant risk factors for the development of a complication in the treatment of open fractures. For example, male sex, age of >60 years, BMI of >40 kg/m^2^, use of nonsteroidal anti-inflammatory drugs, diabetes mellitus, and nicotine consumption are positively correlated with the development of an infection or fracture healing disorder [63]. This also applies to fractures in the context of multiple injuries (polytrauma) or in the context of agricultural accidents [64,65]. The quality of the statistical models is tested with the help of a residual analysis. Sensitivity analyses are carried out to check the stability of the evaluation and the assumptions made, for instance, the effects of changed influencing variables on the result.

As part of the health economic evaluation, the use as well as the costs of health services and absences from work are considered. For this purpose, a cost-effectiveness analysis is carried out whereby the incremental cost-effectiveness ratio will be calculated. The evaluation of routine statutory health insurance data is carried out according to the principles of good secondary data analysis [66], the recommendations of the “Methods for Health Services Research” memorandum [67], and the standards of the German Evaluation Society [68]. The data basis for the evaluation comprises the statutory health insurance routine data, the data from the case file, and the collected data on the health economic quality of life.

#### Work-Related Evaluation

The original item scaling of all 4 or 5 surveys respectively was retained, and all items were compiled into 1 questionnaire. In total, 4 versions of this questionnaire were created, which are identical in content but have been—with relation to the instructions—slightly adapted to the group (treatment group vs control group) and the time of measurement (before vs after the implementation of the new measures). For example, the instruction before treatment reads “Dear participant,...the clinic where you work...will soon start implementing the new measures” (treatment group) and “...will start implementing the new measures in the foreseeable future” (control group), whereas the instruction in the treatment group after implementing the new measures is “Dear participant,...the clinic where you work...is already implementing the new measures.” The questionnaire was implemented, and postcards with a brief explanation and a QR code with a link to each specific questionnaire version were prepared. Interview guidelines and protocol sheets for the semistructured interview were created, and several fact sheets were written to inform the employees about the interview and data protection aspects.

In the practical implementation of ergonomic data collection, the project staff at Steinbeis University are supported by project staff from University Hospital Münster (Universitätsklinikum Münster [UKM]). Project staff from UKM have contact with the clinics and recruit people there to take part in the questionnaire surveys and interviews.

Before the clinics of cohort 1 start implementing the new form of care in project month 7 (Figure 2), both the clinic staff of cohort 1 (treatment group) and the clinic staff of cohort 3 (control group) will be examined with the EXPERT questionnaire in project months 4 and 5 (measurement time Pre). In project months 12 and 13, clinic staff from both cohorts will repeat the questionnaire (measurement time Post). The same procedure will be followed for cohort 2 and cohort 4, 3 and 9 months later, respectively. Project staff from UKM will recruit enough people in the individual clinics for the surveys. Interested employees in the clinics who are or will be involved in the project can find out more about the survey in advance by means of an information text. The UKM project staff receive a questionnaire link as a QR code from the Steinbeis project staff for the corresponding measurement time tailored to the corresponding group and cohort and send this QR code to a contact person in the clinics. The contact persons pass on the QR code to the participants, who scan it on their mobile phones and answer the questions. Participants are requested to do so as soon as possible after receiving the link. The completed questionnaires are automatically stored anonymously on the Steinbeis University server and later evaluated in summary form.

The UKM staff draw attention to the project-related interviews in the clinics that are already implementing the measures. Interested employees can find out more about the interviews by means of an information text and can contact the Steinbeis project staff to make an appointment for the telephone interview. They then receive an email from Steinbeis with the confirmation of the appointment, the interview questions, and the (renewed) promise that their email address and telephone number will be deleted after completion of the project. The transcripts of the telephone interviews are anonymized using a code. The content collected in the interviews will be evaluated in summary form, quantitatively, and qualitatively.

Regarding the topics of workload, work engagement, work-related resources, and readiness for technology, the main effects and interactions between measurement time (before vs after start of the Extremity Board) and group of measures (treatment vs control) are examined via variance analysis; the result is a 2 × 2 design with the factors time (before vs after) and group (treatment vs control group).

Ergonomic aspects of the telemedicine technology are collected and descriptively evaluated at the second measurement point in approximately half of the clinics.

As staff turnover in hospitals is high, it can be assumed that the groups of employees will be composed differently at each time of measurement. Therefore, no repeated-measure design can be applied. The analyses will be performed as balanced grouping factors with the same number of participants in all conditions (before vs after and treatment vs control). The measurements are considered as a grouping factor of a multiple ANOVA.

The evaluation of the repertory grids yields 2D representations of the negative and positive constructs mentioned by the interviewees during the interviews with respect to the different implementation steps, namely, preparation, development of new standards, interdisciplinary collaboration, and therapy implementation.

The combination of a questionnaire that can be used quickly and easily on a mobile phone and a telephone interview with both predetermined, scaled questions and open-ended evaluations according to the repertory grid technique results in a comprehensive picture of changes in pretest-posttest working conditions and of the success factors accompanying the process both in quantitative and qualitative terms. On the one hand, the web-based questionnaire before and after the introduction of the new form of care quantitatively captures influences on workload, work engagement, work-related resources, and readiness for technology and backs them up through the “difference-in-difference” design. On the other hand, central success factors regarding project implementation will be collected both quantitatively and qualitatively and made visible by means of the repertory grid technology as “mental maps” of the employees in the 31 participating clinics.

## Results

The EXPERT project started in June 2022 with the necessary preparations and agreements among all partners involved. Patient data collection started in April 2023. As of June 13, 2024, data from 425 patients have been included. The web-based survey of clinic employees began in July 2023, and the interviews started in February 2024. So far, 146 people have taken part in the questionnaire survey, and 15 people have taken part in the interviews.

## Discussion

### Principal Findings

The aim of the EXPERT project is to improve the treatment of fractures with open soft tissue damage or postoperative complications rapidly and sustainably. To perform optimized multifaceted treatment, we consider the following disciplines an essential part of the interdisciplinary team: trauma surgeons, vascular surgeons, plastic surgeons, specialists for infectious diseases, radiologists, pharmacologists, hygienists, and microbiologists. We think that an interdisciplinary board needs several disciplines to not only enable a complete interdisciplinary treatment approach but also develop interdisciplinary treatment algorithms for infections or provide interdisciplinary prophylaxis in musculoskeletal surgery.

In addition to the evaluation of the clinical outcome, the cost reduction and cost-effectiveness of the new intervention will be evaluated based on claims data provided by 3 major statutory health insurance companies. The focus will be on use of health services and their cost as well as the duration of incapacity for work and HRQoL.

Furthermore, a process evaluation will explore the impact on workload, job satisfaction, and technology acceptance throughout the implementation phase and individual onboarding processes of the caretakers.

### Comparison to Prior Work

The establishment of standardized treatment pathways in the standard care of patients with open fractures and postoperative infections may increase the chances of recovery for those affected and reduce costs. The previous heterogeneity of treatment methods, which is not expedient, can be reduced. Through largely standardized diagnostics and therapy, processes in the clinics can be optimized, and service processes can be standardized. Unnecessary and redundant diagnostics and treatment steps may be avoided. At the same time, the interdisciplinary treatment perspective allows for a more individualized therapy. This study aims to show that this results in a reduction in direct treatment costs and length of stay in hospital as well as a reduction in secondary costs (eg, due to long sick leave after discharge and revisiting practitioners). This is also in line with previous research findings.

Fractures impact function, quality of life, and psychological well-being. An multidisciplinary approach and early diagnosis are crucial for treatment success. Interdisciplinary support and patient-tailored treatment shortens hospital stays and reduces treatment costs, and standardized treatment pathways optimize clinic processes and reduce unnecessary steps [69-71]. In particular, hospital stays and costs induced by fracture-related infections are reduced [72]. The importance of multidisciplinary collaboration is especially effective for patients with hip fractures [73].

### Strengths and Limitations

When measuring the quality of treatment in trauma surgery, several influencing factors must be considered. The type of injury, the extent of anatomical reconstruction during surgery, soft tissue management, perioperative management, and rehabilitation protocols as well as patient compliance have a major influence on the outcome and make evaluation difficult. For our study, we chose 4 outcome measures: the complication rate after initial surgery (nonunion, infection, unplanned surgical revision, and need for prolonged antibiotic therapy), the number of applied antibiotics, the functionality of the injured limb, and HRQoL.

The clinical outcome is examined using a stepped-wedge design, which allows for the statistical consideration of time effects. The design also has practical advantages, such as the easy implementation of a staggered rollout. Among the weaknesses of a stepped-wedge design are time-sensitive recruitment efforts, complex randomization requirements when assigning hospitals to different cohorts or wedges, the assumption of high treatment schedule fidelity, and effects of the observation (the so-called Hawthorne effect) [74].

We choose a difference-in-difference design, a widely used quasi-experimental design in clinical research, to evaluate work-related outcomes. This design eliminates confounding by comparing outcomes before and after treatment. However, the design relies on a parallel-slopes assumption, which is hard to manage and may not always apply [75].

### Conclusions

The interdisciplinary, cross-disciplinary, and cross-departmental coordination of service providers enables comprehensive interface management in the clinics. The perspective of service providers can increasingly focus on holistic, patient-oriented coordination of management processes, which provides patients with the best possible access to individualized therapy in a timely manner. In addition to health economic advantages, the multidisciplinary treatment is expected to improve the quality of the therapy, which is to be proven through clinical follow-up. In addition, it is conceivable to establish specialized outpatient or inpatient treatment centers for patients with open fractures and postoperative infections in which the new diagnostic and therapeutic pathways are competently applied.

## Appendix

Multimedia Appendix 1 Peer review report by the Deutsches Zentrum für Luft- und Raumfahrt e. V. (DLR) - DLR Projektträger - Bereich Gesundheit (Bonn, Germany).

## Abbreviations

EXPERT: Extremity Boards for process optimization, evaluation, risk minimization, and therapy optimization for fractures with soft tissue damage or postoperative infection of the lower extremities in the trauma network
HRQoL: health-related quality of life
*ICD-10*: *International Statistical Classification of Diseases and Related Health Problems, 10th Revision*
JD-R: job demands–resources
UKM: University Hospital Münster
