# Supplementary material for: Improving the Care of Severe, Open Fractures and Postoperative Infections of the Lower Extremities: Protocol for an Interdisciplinary Treatment Approach
Source: JMIR Res Protoc. 2024 Sep 16;13:e57820. doi: 10.2196/57820 (PMC11451582; doi:10.2196/57820)
Supplement: Multimedia Appendix 1 [file resprot_v13i1e57820_app1.pdf]

Evaluation of the project „EXPERT" by the funding authority

1. Original German version
2. English translation (see below)

DLR Projektträger

Deutsches Zentrum für Luft- und Raumfahrt e. V. (DLR)
DLR Projektträger | Bereich Gesundheit
Postadresse: Heinrich-Konen-Straße 1 | 53227 Bonn
Besucheradresse: Heinrich-Konen-Straße 5 | 53227 Bonn
i. A. Dr. Esther Koch| i. A. Tim Schalla
Telefon +49 228 3821-2285 | Telefax +49 228 3821-1257 | esther.koch@dlr.de
http://DLR-PT.de | http://www.gesundheitsforschung-bmbf.de |

Evaluation des Projektantrags „EXPERT" (01NVF21104)

1 Evaluationskonzept

Es ist ein ausführlicheres Evaluationskonzept vorzulegen, das insbesondere den Zugang zu den Patientinnen und Patienten sowie die erforderliche Fallzahl für den primären Endpunkt nachvollziehbarer darstellt.

Bitte überprüfen Sie noch einmal die Fallzahlplanung und den Effekt der Intervention sowie die entsprechende Kalkulation.

Ferner sind die Prozessevaluation und die Methoden der gesundheitsökonomischen Evaluation zu konkretisieren. Bitte gehen Sie darauf ein, wie und ob eine mögliche Integration der summativen Evaluation in die Effektevaluation geplant ist.

Bitte konkretisieren Sie die Darstellungen zur Rekrutierung und der Fallzahlplanung.

Bitte stellen Sie deutlicher dar, wie der primäre Endpunkt definiert und erhoben wird. Bitte beschreiben Sie die Datenerfassung der einzelnen Komponenten anhand von Primär- und Sekundärdaten und deren Verknüpfung genauer.

Bitte konkretisieren Sie die Einschlusskriterien und die Überschneidung mit dem primären Endpunkt bezüglich der Komplikationen, die bei den Patienten vorliegen.

Bitte beschreiben Sie die Methoden der gesundheitsökonomischen Evaluation genauer. Bitte stellen Sie an dieser Stelle auch Informationen zu einem berücksichtigten Routinedatenverzug zur Verfügung.

Bitte konkretisieren Sie die Rechtsgrundlage für die neue Versorgungsform. Sollte zusätzlich zu dem Selektivvertrag nach §140a SGB auch ein Behandlervertrag nach § 630a BGB in Betracht kommen, ist darzustellen wie eine ausreichende Anzahl an Routinedaten für die gesundheitsökonomische Evaluation sichergestellt wird.

Die Durchführung der Therapieempfehlung in der Primär- oder Sekundärklinik ist bereits Teil der Regelversorgung. Die standardisierte Fallübermittlung und die Vorstellung im Extremitätenboard mit multidisziplinärer Therapieempfehlung gehen darüber hinaus. In den Abschlussberichten ist das Potenzial einer Überführung in die Regelversorgung im Sinne einer Folgeabschätzung darzulegen. Bitte gehen Sie hierauf im Evaluationskonzept ein und stellen Sie dar, dass der Hinweis berücksichtigt wird.

2 Kalkulationsgrundlage

Gesundheitliche Versorgungsleistungen:

Bitte stellen Sie dar, wie die Summen im Formblatt zustande kommen und für wie viele Probanden diese berechnet ist. Bitte überprüfen Sie dahingehend auch das Kalkulationsblatt und führen Sie diese Summe im Kalkulationsblatt mit auf.

Es wird vorsorglich darauf hingewiesen, dass die Stellen für das Fallzahl-assoziierte Personal zu prüfen und ggf. anzupassen sind, sollte sich eine Änderung bei der Fallzahl im Projektverlauf ergeben.

Sie schlüsseln auf, wie der Preis pro Leistung zustande kommt. Es ergibt sich eine Pauschale von 92,00 Euro pro behandeltem Fall. Bitte beschreiben Sie, wie hieraus dann die Summen im Formblatt:

- Kostenpauschale für Primärkliniken des Traumanetzwerks (Abrechnungsziffer 1a) 169.622,60 € und
- Kostenpauschale für Verlaufskontrolle - stand. Rückmeldung an KS (Abrechnungsziffer 1.8) 72.494,80 zustande kommen
- und sich daraus dann die Summen im Kalkulationsblatt ergeben.

Bitte schlüsseln Sie, gerne im separaten Tabellenblatt im Kalkulationsblatt genau auf, wie teuer die Leistung pro Person tatsächlich ist und aus welchen einzelnen Posten (Personal bei der Konsortialführung und Gesundheitliche Versorgungsleistungen bei der Konsortialführung, noch weitere Posten?) sich die Summe in Höhe von 3.004.486,39 € zusammensetzt.

Bitte überprüfen Sie die Fallzahl und stellen Sie dar, warum sich diese von den genannten Zahlen in der Projektbeschreibung unterscheidet (Nur Interventionsgruppe?).

Vorlage einer aktualisierten und detaillierteren Zeitplanung für die Fallzahlerreichung. Bitte geben Sie hier die geplante Anzahl der rekrutierten Patienten pro Quartal (Kontroll- und Interventionsgruppe) an.

Bitte nehmen Sie ebenfalls die SOLL und IST Zahlen für die formative Evaluation auf.

Vorlage eines aktualisierten Meilensteinplans für das Projekt. Im Meilensteinplan muss mindestens ein relevanter Meilenstein in jedem Quartal der Projektlaufzeit vorhanden sein. Mit der Vorlage des Meilensteinplans ist zudem ein aktuelles Gantt-Chart zur Arbeits- und Zeitplanung vorzulegen. Der Meilensteinplan, das Gantt-Chart und der Zeitplan zur Fallzahlerreichung müssen widerspruchsfrei übereinstimmen. Die Darstellung der Quartale im Gantt-Chart muss sich auf die Jahre der Projektlaufzeit beziehen, damit der Projektfortschritt und die Meilensteinerreichung nachvollziehbar sind.

Bitte beachten Sie einen evtl. notwendig werdenden Routinedatenverzug. Bitte fügen Sie entsprechende Meilensteine in den Meilensteinplan ein (Einschluss letzter Patient, letzte Leistung, Vorliegen erster Routinedaten, Vorliegen letzter Routinedaten…)

3 Umgang mit Projektverzögerungen

Umgang mit Verzögerungen

Bitte erläutern Sie, wie Sie mit Verzögerungen im Projekt umgehen, welche Verzögerungen sich in der bisherigen Projektlaufzeit kompensieren lassen bzw. wenn nicht, warum sich die Verzögerungen nicht mehr aufholen lassen.

- Bitte nehmen Sie dazu Stellung, ob mögliche Verzögerungen bzw. die Anpassung der Projektlaufzeit Auswirkungen auf den grundsätzlichen Arbeitsplan bzw. auf das Evaluationskonzept haben.

- Nehmen Sie bitte, gemeinsam mit dem Evaluator Stellung zu der Frage, ob auch bei einer Verzögerung alle ursprünglichen Projektziele erreicht werden können bzw. (sofern zutreffend) welche trotz Laufzeitverlängerung nicht mehr zu erreichen sind.

- Bitte geben Sie auch an, ob die Teilprojekte aller Konsortialpartner verlängert werden müssten. Es ist auch möglich, nur einzelne Konsortialpartner zu verlängern (je nach projektbezogenen Arbeitspaketen). Allerdings ist die Laufzeit für die Konsortialführung bis zum Gesamtlaufzeitende zu beantragen, da diese u. a. koordinierende Tätigkeiten für das Konsortium wahrnimmt.

Dokumentation des Umgangs mit Verzögerungen

Geben Sie an, wie die geplante Laufzeit an Verzögerungen angepasst werden kann. Markieren und begründen Sie Veränderungen in den folgenden Aspekten:

- einen aktualisierten Meilensteinplan (im Einklang mit Gantt-Chart, Evaluationskonzept und dem Zeitplan zur Fallzahlerreichung)

- sowie ein aktualisiertes Gantt-Chart.

- einen aktualisierten Zeitplan zur Fallzahlerreichung (Welche zusätzlichen Maßnahmen werden getroffen, um die Rekrutierung positiv zu beeinflussen? Im Zeitplan zur Fallzahlerreichung ist die Transitionsphase einzupflegen.)

- ein aktualisiertes Evaluationskonzept (Bitte begründen Sie die beantragten Änderungen. Bitte erläutern Sie auch, inwieweit die Aussagekraft der Ergebnisse aufgrund der geringeren Fallzahlen ggf. eingeschränkt ist.)

- eine Potenzialanalyse, welche Fallzahlen im Rekrutierungszeitraum noch realistisch erreicht werden können.

English version (automatic translation)

German Aerospace Center (DLR)
DLR Project Management Agency | Health Area
Postal address: Heinrich-Konen-Straße 1 | 53227 Bonn
Visitor's address: Heinrich-Konen-Straße 5 | 53227 Bonn
i. A. Dr. Esther Koch| i. A. Tim Schalla
Phone +49 228 3821-2285 | Fax +49 228 3821-1257 | esther.koch@dlr.de
http://DLR-PT.de | http://www.gesundheitsforschung-bmbf.de |

Evaluation of the project proposal "EXPERT" (01NVF21104)

1 Evaluation Concept

A more detailed evaluation concept should be presented, which in particular presents access to patients and the required number of cases for the primary endpoint in a more comprehensible way.

Please review the sample size planning and the effect of the intervention as well as the corresponding calculation.

In addition, the process evaluation and the methods of health economic evaluation must be specified. Please explain how and whether a possible integration of the summative evaluation into the effect evaluation is planned.

Please specify the descriptions of recruitment and case number planning.

Please be clearer about how the primary endpoint is defined and collected. Please describe in more detail the data collection of the individual components based on primary and secondary data and how they are linked.

Please specify the inclusion criteria and the overlap with the primary endpoint in terms of complications present in patients.

Please describe the methods of health economic evaluation in more detail. At this point, please also provide information on a routine data delay taken into account.

Please specify the legal basis for the new form of care. If, in addition to the selective contract according to §140a SGB, a treatment contract according to § 630a BGB is also possible, it must be shown how a sufficient number of routine data for the health economic evaluation is ensured.

The implementation of the therapy recommendation in the primary or secondary clinic is already part of standard care. The standardised case submission and presentation to the extremity board with multidisciplinary therapy recommendations go beyond this. In the final reports, the potential of a transition to standard care in the sense of an impact assessment must be presented. Please address this in the evaluation concept and make it clear that the information will be taken into account.

2 Basis for calculation

Health care services:

Please explain how the sums are calculated in the form and for how many subjects it is calculated. Please also check the spreadsheet and include this sum in the spreadsheet.

As a precautionary measure, it is pointed out that the positions for the staff associated with the number of cases must be reviewed and, if necessary, adjusted if there is a change in the number of cases in the course of the project.

They break down how the price per service is determined. This results in a lump sum of 92.00 euros per case treated. Please describe how the totals are calculated in the form:

- Lump sum for primary clinics of the trauma network (billing number 1a) € 169,622.60 and
- Lump sum for follow-up - stand. Feedback to KS (billing item 1.8) 72,494.80
- and then the totals in the calculation sheet are derived from this.

Please provide a breakdown, preferably in the separate spreadsheet in the spreadsheet, exactly how expensive the service per person actually is and which individual items (personnel for the consortium management and health care services for the consortium management, other items?) make up the sum of € 3,004,486.39.

Please check the number of cases and explain why they differ from the numbers mentioned in the project description (Intervention group only?).

Submission of an updated and more detailed timeline for the achievement of the number of cases. Please indicate the planned number of patients recruited per quarter (control and intervention group).

Please also include the TARGET and ACTUAL numbers for the formative evaluation.

Submit an updated milestone plan for the project. The milestone plan must include at least one relevant milestone in each quarter of the project lifecycle. The submission of the milestone plan must also be accompanied by an up-to-date Gantt chart for work and time planning. The milestone plan, the Gantt chart and the timeline for achieving the number of cases must match without contradiction. The quarters in the Gantt chart must refer to the years of the project's duration in order to track the progress of the project and the achievement of milestones.

Please note that routine data delays may become necessary. Please include appropriate milestones in the milestone plan (inclusion of last patient, last service, availability of first routine data, availability of last routine data...)

3 Dealing with project delays

Dealing with delays

Please explain how you deal with delays in the project, which delays can be compensated for in the project period so far or, if not, why the delays can no longer be made up.

- Please comment on whether possible delays or the adjustment of the project duration have an impact on the basic work plan or the evaluation concept.

- Together with the evaluator, please comment on the question of whether all original project goals can be achieved even in the event of a delay or (if applicable) which can no longer be achieved despite the extension of the duration.

- Please also indicate whether the sub-projects of all consortium partners would have to be extended. It is also possible to extend only individual consortium partners (depending on project-related work packages). However, the duration of the consortium management must be applied for until the end of the entire term, as it is responsible for coordinating activities for the consortium, among other things.

Documentation of how delays are dealt with

Specify how the scheduled runtime can be adjusted for delays. Highlight and justify changes in the following aspects:

- An updated milestone plan (in line with the Gantt chart, evaluation concept and the timeline for achieving the number of cases)
- as well as an updated Gantt chart.
- an updated caseload achievement timeline (What additional measures are being taken to positively impact recruitment? The transition phase must be included in the timetable for the achievement of the number of cases.)
- an updated evaluation concept (Please explain the reasons for the requested changes. Please also explain to what extent the significance of the results may be limited due to the smaller number of cases.)
- a potential analysis of which case numbers can still realistically be achieved in the recruitment period.
